# The Key Role of Cultural Preservation in Maize Diversity Conservation in the Argentine Yungas

**DOI:** 10.1155/2013/732760

**Published:** 2013-09-03

**Authors:** Norma I. Hilgert, Fernando Zamudio, Violeta Furlan, Lucía Cariola

**Affiliations:** Instituto de Biología Subtropical, CONICET, Facultad de Ciencias Forestales, Universidad Nacional de Misiones, Centro de Investigaciones del Bosque Atlántico, Bertoni 85, 3370 Puerto Iguazú, Argentina

## Abstract

Farmers' decisions on what to grow and why can contribute in understanding the conservation of agrobiodiversity. Culture and ethnicity are indicated as first-class factors leading preservation of heirloom cultivars but this has been little considered in studies examining factors that influence the loss or preservation of agrobiodiversity. We propose that corn's ethnotaxa of less diverse uses, which are also key partners in local cultural reproduction, are usually cultivated by a few households. We analyse if there is a relationship between uses and richness of cultivated ethnotaxa at household level and describe corn's medicinal and ritual uses. We found 25 cultivated ethnotaxa, heterogeneously distributed in the region, and we also found that ethnotaxa with less diverse uses are cultivated in fewer households. We identified that, at regional scale, richness is related with food use diversity. The most frequently cited medicinal uses were urinary and tract infections, diarrhoea, and liver disorders. Medicinal recipes involve combinations with other elements. Maize is an indispensable resource in the rituals that propitiate productive activity, to augur prosperity or misfortune according to signals. We have identified the vulnerability in preserving the richness of corn in the region and the factors that shape its cultivation at different scales.

## 1. Introduction

Conservation and loss of agrobiodiversity is a concern to academics, development institutions (governmental or not), and to local populations [1–4], facing the modernization, socioeconomic, and environmental changes that occur both globally and regionally. Migration, pests outbreaks, and climate changes, among other variables, promote transformation in the composition and dynamics of agricultural parcels, changes that ultimately alter the functionality of contemporary rural landscapes. These changes occur in cascade through complex mechanisms (top-down and bottom-up) and disseminate simultaneously in different directions and scales [5, 6].

Farmers' decisions on what to grow and why can contribute to understand the conservation of agrobiodiversity and can help scientists to realize farmers' role in this process [2, 4, 7].

Until not long ago it was held, from academia, that the decisions of local people were guided by economic, agronomic, and ecological variables (see review in Veteto [8]). At present, culture and ethnicity are recognized as first-class factors leading conservation of heirloom cultivars [8–12]. Among the driving factors behind agrobiodiversity, persistence cultural salience has been identified in the first place and utilitarian salience in the second. The first concept is understood as the selection criteria that are specifically related to culturally defined preferences and influences such as foodways, cultural heritage, and memory; instead, the second one is understood as the selection criteria that are specifically related to market value, productivity, environment adaptation, and resistance [8, 13–16]. 

The assessment of criteria or the decisions that shape local conservation of agrobiodiversity is central to guarantee people's food sovereignty and generation of *in situ* conservation plans which involve local peasants. Analytic categories used to classify corpus of the local knowledge (religious, economic, alimentary, medicinal) are abstractions of a matrix of interconnected elements that, analyzed together, explain agricultural decision making and agrobiodiversity persistence [8, 17]. 

Indeed, the use of resources may differ depending on the context or the occasion: daily consumption of food within the household, use in collective festivities, offering to supernatural entities, and the act of sharing food with ancestors, among others [18, 19]. In this context, a given resource: alimentary, medicinal, material can officiate as a symbol understood as an object represented by general consensus or something evocative [20].

The approach of this paper is supported by previous research in the study area, which found that the local health concept that matches the Andean concept is understood as an holistic balance of a person environment [21–25]. According to our experience, to analyze cultural driving factors behind persistence of agrobiodiversity, it is necessary to develop a comprehensive analysis of the role of corn in the lives of the Yungas residents. For this reason, we considered the general uses of maize and the uses of herbal medicine which involves maize varieties in particular, the ritual uses of maize (e.g., meals prepared in agricultural celebrations or funerals), and those practices that reflect protective value assigned to maize for being the legacy of La Pachamama, the principal chthonic deity of the region.

In the region of our study, a decrease in agrobiodiversity, the gradual abandonment of different production spaces, and the replacement of some species and varieties by others as an adaptive response to these changes in production have been described [26]. In relation to this, and bearing in mind that the selection criteria affect the richness of grown ethnotaxa at household and regional levels, we propose that ethnotaxa of less diverse uses, which are also key partners in local cultural reproduction for their medicinal value, symbolic and/or ritual, are usually cultivated by a few households. This is consistent with those who assigned a leadership role to ritual values for conservation of agrobiodiversity [27, 28]. To evaluate this, we analyze if there is a relationship between the uses assigned to each ethnotaxon and the richness of cultivated ethnotaxa at household level and describe the medicinal and ritual uses of maize in three regions of the Argentine Yungas.

## 2. Background

The inhabitants of the communities under study are descendants of Andean cultures, Chaco, and Hispanic who have converged historically and culturally [29]. Due to its geopolitical history and geographical location in the Baritú region, unlike the other two, there are established social and commercial ties with Bolivian communities [30]. At present they identify themselves as *criollos*, although this name also identifies people of different cultural roots. They maintain a set of practices and beliefs, among which the cult of the Pachamama, a characteristic element of Andean cultures, stands out, as mentioned above. They also profess Catholicism and Evangelism (the latter more recent and in current expansion). The Spanish language is enriched with Quichua and Guaraní terms (to a lesser extent) [31].

Regional economy is based on a system of shifting agriculture, transhumance, and gathering, to a lesser extent. Shifting agriculture and transhumance which consists on the use of different cultivation and cattle breeding areas along altitudinal gradients favour vertical use of the environment. Each family moves periodically along the year to the *cerro*, *valle,* and *monte*, where they cultivate and pasture their animals [26]. However, at present, transhumance in some areas has been reduced and abandoned in others [32]. This production system is usually complemented with paid work carried out within or outside the community in nearby settlements or villages and allowances granted by the government have proven to be a useful contribution to household resources. Local women also weave wool blankets, saddlebags, and crafts for their own households or for sale, as an other mean of getting some income. Nowadays, they are increasingly experimenting dyeing plant species in search for new colours and combining different species together, which is a new phenomenon [33]. 

The most important crops are *Zea mays* (Poaceae) and *Solanum tuberosum* (Solanaceae). In each altitudinal belt, different varieties of the same species are produced, together with *Cucurbita *spp. (Cucurbitaceae) and several varieties of *Phaseolus vulgaris* (Fabaceae). The currently cultivated crops include old maize landraces, recently incorporated foreign elements, and mixed product of hybridization between some of the above mentioned [26, 34].

Regarding local medicine, the current ethnomedical system involves, according to the classification proposed by Molina [35], practices such as traditional, homemade, self-treatment, religious, and biomedicine practices. As appropriate, health practices are carried out by the rural doctor who has no formal education or degree of specialization, or by people with graduate studies or degrees, as the local doctor and the nurse at the sanitary post.

Local people recognize three origins for ailments: natural, sociocultural (i.e., when a food taboo is not respected), and supernatural (i.e., witchcraft, air diseases, templar diseases) [32, 36, 37]. The last two types can be diagnosed and treated only by a rural doctor (called *curandero* locally). Diseases of natural origin, like cough, dyspepsia, headache, and postpartum pain, are usually solved within the family environment and it is not necessary to have any formal training or carry out any ritual process [36]. The diagnosis is made by the rural doctor, based on an interview with the patient; other techniques may be also applied. These techniques agree in general with the divinatory practices described by Amodio [38]; they include the “reading” of *Erythroxylum coca* leaves, the “reading” of the urine, the *pulseo* (or diagnosis by the pulse), the *alumbriada*, and rubbing of a body with alum which is later burnt to interpret the ashes, among others. The latter consists of simultaneous diagnosis and treatment, as the affection is considered to be transmitted to the mineral and destroyed through burning [30, 36, 37].

## 3. Material and Methods

**Figure 1:**
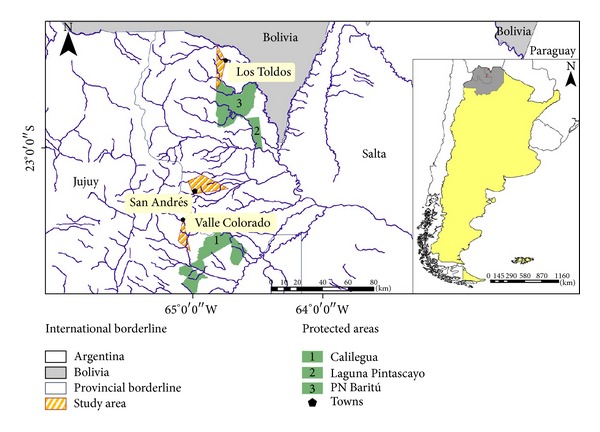
Studied area.

The study area covers three groups of settlements: settlements located in the surroundings of Baritú National Park (Deptartment Santa Victoria, Salta), settlements located in the former large farm of San Andrés (Deptartment Oran, Salta), and those above the Calilegua National Park (hereinafter Los Toldos, San Andres, and Valle Colorado) (Figure 1). The sampling unit was the nuclear family. Households were randomly selected within the group of people willing to participate in the study.

Data were collected in three steps, covering a total of 118 informants (in 150 surveys). Visits to the study area were carried out from 1994 to 2000 on the first stage of the investigation, at that moment global ethnobotanical practices of local medicinal knowledge were summarized in 59 surveys; the second stage visits took place from 2006 to 2008 accomplishing a total of 91 surveys; the third stage was made in 2012 and 10 surveys were carried out. In the first stage of the project, a semistructured interview was directed to the domestic uses of maize. In subsequent stages, the interview deepened in different aspects of local maize varieties in each household (richness of cultivated ethnotaxa and assigned uses). In particular, the respondents were asked about medicinal and ritual uses of maize, as well as the plants they used to combine with it, the plant parts used, the methods of preparation and administration, dosage, treatment time, and illness duration. We took also into account observations made in festivals and religious and propitiatory rituals developed in the context of agricultural labor.

The plant specimens were collected in the presence of the study participants. Then, voucher specimens were identified by Norma Hilgert and were deposited in the herbarium of the Museo de Ciencias Naturales of the Universidad Nacional de Salta, Argentina. The nomenclature used follows Flora del Cono-Sur (http://www2.darwin.edu.ar/Proyectos/FloraArgentina/FA.asp) and Tropicos (http://www.tropicos.org/Home.aspx) data base. 

The majority of the collected maize varieties were identified by Cámara Hernández, researcher of the Agronomical Faculty of the National University of Buenos Aires, and the remaining others were identified by the authors. The collected samples were deposited in the Banco de Germoplasma del INTA Pergamino (Argentina).

Regarding race and/or varieties of maize, in this paper we consider maize populations distinguished by farmers as landraces according to the proposal by Perales et al. [11], populations that are often known locally as *criollos*, indicating that it is local corn, while all landraces which have local name in the region are hereinafter referred to as ethnotaxa. We considered only those ethnotaxa that have been cited by more than 5 people. In such ethnotaxa, besides vernacular name, they recognize special characters (such as colour, cooking, and/or medicinal properties and plant size). Moreover, under the category of commercial maize, there is a pool of different ethnotaxa that were marked as purchased in markets (e.g., hard corn, yellow corn, yellow corn Abajeño, Cuban Corn, and Corn mule). Other ethnotaxa considered here, as the *overo* or *chesgua* among others, although identified as hybrids by taxonomists, were considered a taxonomic unit in interviews because there was uniformity of criteria at selecting and naming them and in their usage. 

Two variables were measured at household level: richness of maize grown at present (i.e., number of grown ethnotaxa in each household) and diversity of cited uses. Maize uses were grouped in three general categories, alimentary, fodder, and medicinal/ritual. The latter group includes citations of uses of preparation of medicaments, preparation of ritual food, ritual practices, and/or propitiatory practices (such as a particular cultivation of an ethnotaxon as other protective element of crops or housing). Considering these categories, we estimated the diversity of use by ethnotaxa (i.e., number assigned to each ethnotaxon use as food, fodder, and medicinal/ritual, resp.). 

To analyze whether there is a relationship between the ethnotaxa with less diverse uses and the number of households that cultivate them, we performed a diverse Spearman correlation between the true diversity of uses of each ethnotaxon as ln⁡*D* = (Shannon-Wiener index) in García-Morales et al. [39] and the number of households where each ethnotaxon is grown. To identify the types of uses that model the richness of cultivated ethnotaxa, we performed a multiple regression using the richness of crops by household as dependent variable and the number of uses assigned to each ethnotaxon by household for categories food, fodder, and medicinal/ritual as independent variable. In both cases, we used Excel spreadsheets and StatSoft, Inc. [40]. 

For more details on medicinal and therapeutic conditions named in the current paper, see Hilgert [36] and Hilgert and Gil [37] where a thorough analysis of ritual aspects can be found. Finally, more details of culinary preparations named in the present paper can be found in Hilgert [41].

## 4. Results

We have found 25 ethnotaxa of cultivated maize, heterogeneously distributed in the region, 23 in Valle Colorado, 19 in Los Toldos, and 12 in San Andres. There is a low average of ethnotaxa cultivated by a household: 1.2, 2.3, and 3, respectively. Also the range of ethnotaxa grown in each location (1–10, 1–5, and 1–17, resp.) reflects an unequal distribution of varieties within each locality.

When analyzing whether there is a relationship between the ethnotaxa with less diverse uses and the number of households that grow them, at regional scale we found (considering the three areas together) that ethnotaxa with less diverse uses are grown in less households (coefficient: 0.888 (*P* < 0.001)). The same relationship was found locally (Los Toldos: Coef.: 0.908, *P* < 0.001; San Andrés: Coef.: 0.638, *P* < 0.05; Valle Colorado: Coef.: 0.816, *P* < 0.001). This is consistent with the proposed hypothesis.

Moreover, we identified the types of uses that model ethnotaxa richness of households cultivated in different situations. We found that richness of ethnotaxa cultivated by a household is related (adjusted *r* ^2^: 0.626, *F*(3.104) = 60.715, *P* < 0.001, ES: 2.219, *β*: 0.788, *P* < 0.001) to food uses diversity at regional scale.

For settlements in Los Toldos, richness of ethnotaxa cultivated by a household is related (adjusted *r* ^2^: 0.739, *F*(3.39) = 40.722, *P* < 0.001, ES: 1625) to food uses diversity (*β*: 0.602, *P* < 0.001) as fodder (*β*: 0.264, *P* < 0.01) and in medicinal/ritual (*β*: 0.240, *P* < 0.05). 

For settlements in San Andrés, richness of ethnotaxa cultivated by a household is related (adjusted *r* ^2^: 0.304, *F*(3,39) = 7.136, *P* < 0.001, ES: 1.268) to the diversity of uses as fodder (*β*: 0.365, *P* < 0.01) and food (*β*: 0.375, *P* < 0.05). 

Finally, for settlements in Valle Colorado, richness of ethnotaxa cultivated by a household is related (adjusted *r* ^2^: 0.932, *F*(3.28) = 142.69, *P* < 0.001, ES: 1.477, *β*: 0.937, *P* < 0.001) to diversity of food uses.

In relation to its medicinal uses, maize is often used as the only component in preparations, but the most common recipes involve its use in combination with other plants and/or different resources. These mixtures include the use of two or more species. We registered the use of 25 plant species, belonging to 18 families. The affections most frequently treated were urinary infections (6 different recipes), diarrhoea and liver disorders (3); for *limpias* and *alumbriadas*, for fever, and to avoid air and cold diseases (2); against *cangrena* (intensive urinary pain caused by hot imbalance), against general pains, as dietary supplement, as a stimulant, to heal pimples, and to remove the placenta (1). Toasted flour and/or stigma are used in different modes of preparation administrated orally (infusions, decoctions, masticatory, inhalation, and foods) and in topics (poultices, rubbing, and ointment) (Table 1). Indistinct ethnotaxa is generally used in these preparations, except for some ailments, or particular cultural practices, described in the following section. 

## 5. Maize Symbolism

**Figure 2:**
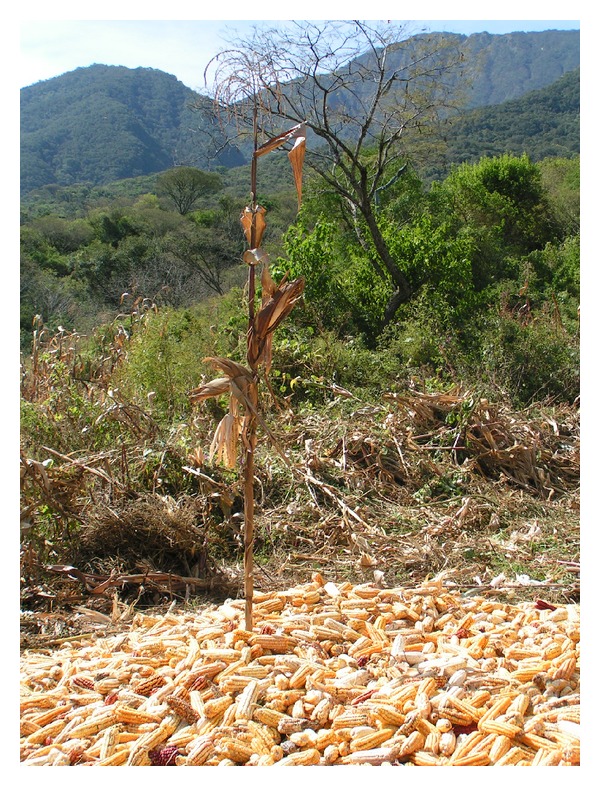
In this picture the e*ra* of cobs is observed. The complete upright corn plant in the center of this mound represents the *Pachamama. *

In the study region, maize is considered the representation (or materialization) of the *Pachamama*, and it is an indispensable resource in the rituals that propitiate or thank the productive activity, for example, (1) in carnival dances, where local people brandish maize plants as a flag or handkerchief and (2) in *San Isidro Labrador* (patron of farmers) festivity in mid-May, which celebrates the harvest and calls for work and prosperity in future harvests. On this saint day, a pilgrimage is performed from the temple to the household responsible for the cornfield celebration. There, in the field, the image of the saint is placed on the altar prepared for this particular purpose. In the vicinity of the altar, the cobs are stacked cropped (in a mound called *era*) in its middle and one maize plant is left standing with two corn cobs. During the celebration, around that *era*, people dance and sing, in gratitude for the production (Figure 2).

*Culli* landrace has a particular assigned property: the ability to protect the cornfield and housing. In the first case, the *culli* is always grown in a plot—this sector can be in the middle, a cross side, or somewhere else in the cornfield, in order to prevent the winds and summer storms from pulling down the cornfield (process called locally as *el volteo de la chacra*). *Culli* is more often cultivated for its benefiting action rather than for its culinary properties. The surface of the* culli* cultivated by each household is small in relation to its total production of corn. Moreover, when it is used for symbolic protection of the housing, it is hanged on the outer frame of the kitchen door*∖*as an *eraquita* or *simbita* that is to say as a pair of cobs tied through the braided husk.

In addition, the listed benefactor properties are the underlying use of this ethnotaxon in *limpias *and* alumbriadas* and in preparing *chicha* (fermented beverage made from corn) to be used in ritual contexts. A *limpia* is a curative procedure that involves scrubbing the patient's body with a mixture of *coca, tabaco, culli* cornmeal, and alum (see Table 1). In this case, this ethnotaxon is considered capable of providing protection to repel or remove *bad air,* witchcraft, and *coldness* (seen from templar medicine) of the patient's body.

It was also recorded that *culli's *strength to scare the *bad air* and misfortunes probably comes probably from its black or dark colour. However, its dark colour is often mentioned as an undesirable feature for everyday kitchen use (except for the *chichi*; see below), as it is considered an unpleasant ingredient which turns everything black.

**Figure 3:**
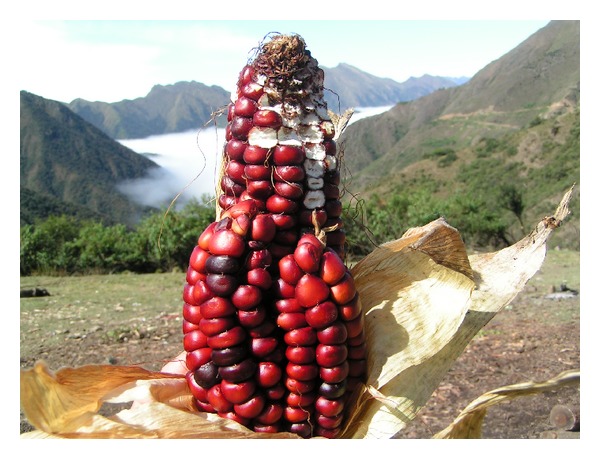
In this picture an unusual cob corn is observed, called *Pachamama* or *Sara.* It is interpreted that the presence of these structures in a cornfield predicts good harvest in the coming years.

Another particular feature assigned to maize is the capacity to augur prosperity or misfortune, according to certain signals. Among the positive signs is the appearance in the cornfield of unusual cobs with more than two cobs together (like a basket) (Figure 3). These cobs are called *Pachamama* or *Sara* and are interpreted as Mother Earth and her children. If there are only two cobs together, one larger than the other, it is interpreted as a representation of the *semillera*, which is the woman in charge of seeding. The explanation of local people is that the cob is the representation of the mother with the baby carried on the back (as mothers carry usually their young children in the region). It is interpreted that the presence of these corns in a cornfield predicts good harvest in coming years. These rare cobs are stored in a special place in the house without removing the husk that is with *la ropita de la Pachamama *(the clothes of *Pachamama*). 

Among the negative signals, the infection of corn plants with *musura* (*Ustilago maydis*) is frequently mentioned. It is believed that when this fungus appears in the cornfield it is a sign that a family member will die. To avert the omen, infected plants are removed and pigs are fed with them, a practice that also prevents the spread of the fungus.

Finally, in all religious rituals or celebrations, corn is prepared in different meals and serves to entertain people, for the deceased (on the feast of *Todos los Santos*), and *Pachamama*, including the following.

- *Chicha*. The use of 12 ethnotaxa was registered, with the highest number of citations for *culli* and *morocho*. In the case of *culli* landrace, since the preparation of this drink requires a lot of flour, when the available volume is scarce, medicinal preparation or any of the other ritual dishes is given priority rather than *chicha*.
- *Tistinchas*. It is a stew prepared with cobs, for which three alternate rows of grains are extracted (corn used is *morocho*, *blanco boliviano*, *overo*, *tucumano,* and *colorado*). Lamb or beef are added, as well as broad beans (*Vicia faba*), beans (*Phaseolus *spp), peppers (*Capsicum* spp), green potatoes (*Oxalis tuberosa*), and potatoes (*Solanum tuberosum*), among other ingredients. The preparation is boiled overnight and prepared especially for the first of August, the day of the *Pachamama*.
- *Pire *(or* Piri*). It is prepared from roasted cornflour, water, and onion; the last one is fried with abundant oil or fat. The dish is a kind of soup. It can be seasoned with salt, sugar, or both. *Culli* corn is employed, together with *sauceño *and *overito*. It is prepared especially for the day of the *Pachamama*. That day, at dawn, the *pire* is placed on the roof of the kitchen in order to feed the birds. On that day, approaching birds are considered mythical beings, able to intercede for the good fortune of households which have offered up.
- *Tulpo.* It is a Type of stew made with cornflour and locally produced vegetables (similar to *tistinchas*). *Morochos* and *maíz blanco* are preferred. This dish is prepared especially for summer parties (Christmas and Carnival).

## 6. Discussion

In our study area, the maize is present in different contexts of family life, community ritual life, and the agricultural cycle. In the literature, there are numerous examples with a similar picture to the one found here, where the same resources are redefined as everyday food, medicine, and ritual resource in collective festivities and offerings [3, 18, 21, 42–44].

The observed relationship between the diversity of uses and the number of households that cultivate them can be interpreted as an indicator of regional vulnerability of conservation of less versatile ethnotaxa. This is consistent with the asymmetry in the number of cultivated ethnotaxa per family and the high number of very rare ethnotaxa found by Velásquez-Milla et al. [3] in Andean towns in Peru. Our records also partially match with those observed in neighboring communities by Pinotti et al. [45]. These authors noted a positive relationship between factors such as diet and conservation of local resources, like us, but identified the informal trade and exchange networks as the main means of obtaining different corns, to the detriment of local agriculture.

We found that at the regional level alimentary uses are the ones which influence mostly the cultivation of ethnotaxa, so their conservation is strongly associated with the traditional cuisine conservation. This aspect has been pointed out previously in numerous occasions. Díaz et al. [46] define maize and potato as diacritic elements of present regional food (both traditional and tourism-related innovations). In line with the diversity of food uses found in the present, Cámara Hernandez et al. [34], Velásquez-Milla et al. [3], and Díaz et al. [46] explain the preferential and alternative uses of ethnotaxa in different preparations, consistent with the particular characteristics of each ethnotaxon (oily, mealy, hard, or soft, etc.).

Instead, we found that local crop modellers of ethnotaxa vary by region. We have noted that Los Toldos is the only region in which the medicinal and ritual uses have significant importance in the conservation of ethnotaxa. This could be interpreted as an indicator of greater conservation of medicinal and ritual practices associated with corn, although it could also be a result of the long association between villagers and researchers, which facilitates the obtention of information not typically shared with outsiders, in accordance with the experienced by Pinotti et al. [45] in neighbouring regions.

In San Andres for a change, the use of maize as a fodder has the principal application among the cultivated ethnotaxa. This is probably due to the changes in land use experienced during the last 20 years. It is specially evident in the abandonment of the vertical use of the environment and the slow abandonment of transhumance [26, 43].

Regarding the medicinal applications, it has been observed that the corn is used in many recipes that include other plant species (Table 1). In these recipes, we observed indiscriminate frequent employment of marketed cornstarch or flour of any ethnotaxon. This suggests that the corn is used as a binder or thickener, rather than for its medicinal properties. In other regions, this flexibility and replacement of products in medicinal preparations have been recorded as common in domestic medical practice and is generally associated with the availability of products [47–49]. Therefore, it is important to conduct in the future more investigations on the role assigned to different resources to prepare medicines and identify the ethnotaxa with preferential medicinal use and also those which function as replacement alternatives for the most important ones. In respect to the protective or propitiatory role assigned to corn *culli*, similar uses have been cited for other ethnotaxa in Mexico [50].

In relation to the positive (in the case of medicinal/ritual uses) and negative (in everyday culinary uses assigned to *culli* due to its black colour) assessments, at present there is increasing evidence of the importance of sensory perception when selecting resources, both cultivated and medicinal [3, 51–54]. According to Leonti et al. [55] and Boster [56], these characteristics also officiate as mnemonic resources for the social transmission of knowledge and selection criteria. Undoubtedly, in the case of maize, visual appearance of ethnotaxa is an essential tool when selecting seeds for the next planting, although further studies would be necessary to define what other aspects are involved. 

## Figures and Tables

**Table 1:** Medicinal corn uses.

Species, Family (herbarium)	Local name	Use	Administration
*Adesmia inflexa *Griseb., Fabaceae (1224; 1714)	*añagua *	To remove the placenta	A handful of roots is boiled in 5 L of water with about 5 cm of *Cortaderia selloana* roots. Seven successive poultices are placed in the back with this mixture next to a hot corn cob tied with a black cloth. Each application is left until it gets cold. This treatment is completed covering the back with two warm saddle cloth (one heated in the stove and the other in the back of a horse)

*Artemisia absinthium* L. (2433); *Tanacetum parthenium* (L.) Sch. Bip. (1479), Asteraceae	*carqueja *	Against urinary affections	An infusion is prepared in half a liter of water with three or five fresh leaves, combined with a piece of *Equisetum bogotense *(or* E. giganteum*), three fresh leaves of *Pluchea sagittalis*, and a tablespoon of corn flour. It is drunk several times for several days
		Against diarrhea	A decoction in half a liter of water with three leaves mixed with three leaves of *Erythroxylum coca* var. *coca *a piece of *Cinnamomum zeylanicum, *and handful of seeds of *Pimpinella anisum *is prepared. After being withdrawn from the fire, a little of cornflour is added. It is drunk lukewarm or cold several times for several days until symptoms disappear

*Cinnamomum zeylanicum* Blume, Lauraceae (1575; 2330)	*canela *	Against diarrhea	See full recipe under *Artemisia absinthium *
		Against hepatic affections	A piece is boiled in 1 L of water; after being withdrawn from the fire, a little of flour is added. It is drunk lukewarm or cold several times for several days until symptoms disappear

*Citrus x limon* (L.) Osbeck, Rutaceae (1590)	*limón *	Against fever	A few drops of juice are mixed in warm water with a spoon of corn flour and a handful of ground flax (*Linum usitatissimum*) seeds. It is drunk several times until symptoms disappear

*Citrus x sinensis *(L.) Osbeck, Rutaceae (2053)	*naranja, n. dulce *	Against hepatic affections	A few drops of juice are mixed with a spoon of corn flour in warm water. It is drunk several times for several days until symptoms disappear
		Against diarrhea	See full recipe under *Artemisia absinthium *

*Cortaderia selloana* (Schult. and Schult. f.) Asch. and Graebn., Poaceae (1722)	*cortadera *	To remove the placenta	See full recipe under *Adesmia inflexa *

*Dolichandra unguis-cati* (L.) L.G. Lohmann, Bignoniaceae (2015, 2192)	*uña de gato *	Against urinary affections	A piece of the plant with the stigmas of two spikes, one or two roots of *Plantago australis*—or *P. major* or *P. myosurus*—a piece of *Equisetum bogotense* (or E. giganteum), and handful of ground flax (*Linum usitatissimum*) seeds are boiled. When it reaches the boiling point, add half grated potato (*Solanum tuberosum*). It is drunk lukewarm or cold several times for several days until symptoms disappear

*Equisetum bogotense* Kunth, (1394); *E. giganteum* L. (1617), Equisetaceae	*cola de caballo *	Against urinary affections	See full recipe under *Adesmia inflexa *
		See full recipe under *Dolichandra unguis-cati *
		A piece of the plants combined with the stigmas of two spikes is boiled in 500 mL of water. It is drunk at own discretion, as a cold soft drink during the day

*Erythroxylum coca* Lam. var. *coca*, Erythroxylaceae (2108)	*coca *	For* limpias* and *alumbriadas *	An ointment is prepared with leaves of *acullico*—insalivated coca—and cornflour of *culli* roasted. This must be rubbed in the patient's body and burn it. If treatment is *alumbriada*, then to this paste alum is added. This metal, when burned, takes the form of the element that is causing the condition and orient to doctor about the treatment

*Iresine diffusa *Humb. & Bonpl. ex Willd., Amaranthaceae (1897)	*sacha arborito *	As dietary supplement, as a stimulant	The grounded ashes of the burned branches are mixed with mote, resulting in a paste known as lye. This product is used during the coca leaves insalivation, known as *coqueo *

*Linum usitatissimum* L., Linaceae (1621; 2317)	*linaza *	Against fever	See full recipe under *Citrus limon *
		Against urinary affections	See full recipe under *Dolichandra unguis-cati *

*Nicotiana tabacum* L., Solanaceae (1474; 1487)	*tabaco *	For* limpias* and *alumbriadas *	A cigar is prepared with dry crushed leaves of snuff bundled in corn husks and smoked with the smoke exhale on the body of the patient during the treatment

*Pimpinella anisum* L., Apiaceae (manufactured product)	*anís castilla *	Against diarrhea	See full recipe under *Artemisia absinthium *

*Plantago australis *Lam. (2534); *P. major *L. (2439); *P. myosurus* Lam. (2222), Plantaginaceae	*llantén *	Against urinary affections	The stigmas of two spikes are boiled in 500 mL of water with two fresh leaves. It is drunk at own discretion, as a cold soft drink during the day
		See full recipe under *Dolichandra unguis-cati *

*Pluchea sagittalis* (Lam.) Cabrera, Asteraceae (2392; 2571; 2530; 2179)	*cuatro cantos *	Against urinary affections	See full recipe under *Artemisia absinthium *

*Prunus amygdalus* Batsch, Rosaceae (manufactured product)	*almendra *	Against fever	An ointment with a few drops of almonds oil is mixed with a spoon of cornflour in warm water. It is put in the brow until fever disappear
		To heal pimple	*Mote*—corn—and seeds of almonds are crushed. This ointment is placed on the pimple

*Punica granatum* L., Lythraceae (2469);* Passiflora tenuifolia* Killip, Passifloraceae (1515)	*granda, granadilla *	Against diarrhea	An infusion is prepared with two or three pieces of the skin of the dry fruit, with two spoonfuls of flour in 250 mL of water. It is drunk lukewarm once or twice; if symptoms persist, the treatment is repeated for two consecutive days

*Sambucus nigra* L. subsp. *peruviana* (Kunth) R. Bolli, Adoxaceae (2142)	*mololo *	Against* cangrena *(intensive urinary pains caused by hot imbalance)	Two or three flowers are boiled in 500 mL of water. A spoon of corn flour and honey is added. It is drunk at own discretion, as a cold soft drink during the day

*Solanum tuberosum* L., Solanaceae (1924; 1933; 1907)	*papa *	Against urinary affections	See full recipe under *Dolichandra unguis-cati *

*Origanum x appli *(Domin) Boros, Lamiaceae (1448; 2242)	*orégano *	To avoid* air* and cold diseases	During the puerperal, it is advisable for the mother to eat corn-based foods seasoned with oregano. This is done to avoid coldness and the entrance of air which could cause a general weakness

*Zea mays *L., Poaceae (1386; 1711; 2423; 2424)	*maíz *	For all diseases cited above	See full recipes under all species above mentioned
		Against general pains	The cornflour is mixed with egg yolk, salt, and pork fat or chicken excrement. It is used as an ointment in different painful parts of the body
		Against hepatic and urinary affections	The stigmas of two spikes are boiled in 500 mL of water. It is drunk at own discretion, as a cold soft drink during the day
		For templar imbalances manifested in urinary affections	See recipe above mentioned
